# Promoting lifestyle medicine research in Ghana: lessons learned from Centre for Lifestyle Medicine and Behaviour (CLiMB) Ghana hybrid workshop

**DOI:** 10.3389/fpubh.2025.1636462

**Published:** 2025-09-03

**Authors:** Hibbah Osei-Kwasi, Daniel Boateng, Sawudatu Zakariah-Akoto, Alice Ojwang, Faith Agbozo, Emmanuel Assasie, Phyllis Addo, Maame Yaakwaah Blay Adjei, Victor Mogre, Robert Akparibo, Richmond Aryeetey, Andrew Levy, Brenda Abu, Sophia D. Amenyah, Dorotheah Obiri, Sandra Kushitor, Maria Inês Varela-Silva, Paula Griffiths, Amanda J. Daley

**Affiliations:** ^1^Centre for Lifestyle Medicine and Behaviour, Loughborough University, Loughborough, United Kingdom; ^2^Department of Epidemiology and Biostatistics, Kwame Nkrumah University of Science and Technology, Kumasi, Ghana; ^3^Department of Global Public Health and Bioethics, Julius Center for Health Sciences and Primary Care, University Medical Center, Utrecht University, Utrecht, Netherlands; ^4^Nutrition Department, Noguchi Memorial Institute for Medical Research, University of Ghana, Accra, Ghana; ^5^Department of Health and Biomedical Sciences, The Technical University of Kenya, Nairobi, Kenya; ^6^Department of Family and Community Health, Fred N. Binka School of Public Health, University of Health and Allied Sciences, Hohoe, Ghana; ^7^Department of Physical Education and Sport Studies, University of Ghana, Accra, Ghana; ^8^Department of Nutrition and Food Science, School of Biological Science, College of Basic and Applied Sciences, University of Ghana, Accra, Ghana; ^9^Department of Health Professions Education and Innovative Learning, School of Medicine, University for Development Studies, Tamale, Ghana; ^10^School of Medicine and Population Health, Division of Population Health, Sheffield Centre for Health and Related Research, University of Sheffield, Sheffield, United Kingdom; ^11^School of Public Health, University of Ghana, Accra, Ghana; ^12^Department of Psychology, Edge Hill University, Ormskirk, United Kingdom; ^13^College of Health Sciences and Technology, Wegmans School of Health and Nutrition, Rochester Institute of Technology, Rochester, NY, United States; ^14^Department of Sport, Exercise and Rehabilitation, Northumbria University, Newcastle upon Tyne, United Kingdom; ^15^Department of Immunology, Noguchi Memorial Institute for Medical Research, College of Health Sciences, University of Ghana, Accra, Ghana; ^16^Department of Community Health, Ensign Global College, Accra, Ghana; ^17^School of Sports, Exercise and Health Science, Loughborough University, Loughborough, United Kingdom

**Keywords:** nutrition, lifestyle, physical activity, Ghana, non-communicable disease, medicine, research, dietary behavior

## Abstract

The rapid rise in urbanization across many African countries is contributing to the increasing prevalence of non-communicable diseases (NCDs) in both adults and children and presents a significant challenge to health services across the continent. In May 2023, a 2-day workshop was held in Ghana, coordinated by the Centre for Lifestyle Medicine and Behaviour (CLiMB) at Loughborough University and in collaboration with partners from three Ghanaian Universities. The workshop brought together academics, clinicians, public health specialists and civil society organizations from Ghana, UK, France, the Netherlands, the USA, and Kenya. Our main objective was to identify scalable lifestyle interventions to address the growing burden of NCDs in Ghana. The 2-day workshop focused on identifying salient research priorities for the prevention of NCDs. The immediate research priorities outlined were (1) conduct observational research in Ghana to identify feasible dietary and physical activity changes to reduce NCDs; (2) undertake a Delphi Study to -prioritize a research agenda on NCDs, focusing on lifestyle behaviors and involving researchers, policymakers, and implementers; (3) perform a comprehensive mapping and realist synthesis of diet and physical activity interventions, policies, and programs in Ghana and across Africa to assess their effectiveness and relevance; and (4) evaluate contextual factors influencing physical activity participation in the regions of Ghana and Kenya. Workshop participants highlighted the importance of adopting a multidisciplinary research approach and emphasized the critical need for strong collaboration among all stakeholders, including the public, for implementing effective lifestyle interventions to improve the health of Ghanaians.

## Background

The high prevalence of non-communicable diseases (NCDs) in many low- and middle-income countries (LMICs), including those in Africa, presents a significant challenge to health services because infectious diseases are still prevalent, and costly to screen and treat. Of all NCD deaths globally, 77% occur in LMICs (1–4). In Ghana, for example, NCDs were responsible for 43% of total deaths in 2016 (4, 5). Until the past decade, however, NCDs were perceived as conditions mainly affecting high-income countries. As a result, research on the incidence, prevalence and associated risk factors of NCDs in African nations remains limited. Therefore, further research is required to support the adoption and implementation of health behavior change policies and interventions to address NCDs issue in African countries.

Over the past decade, NCDs have emerged as a significant public health challenge in Ghana and across Africa (3). This increase in prevalence is attributed to several factors, including rapid urbanization, the nutrition transition (changes in dietary patterns) leading to unhealthy dietary patterns, sedentary lifestyles, air pollution, tobacco smoking, and drinking alcohol (6). Consequently, there has been a notable increase in the development of national policies and initiatives indicating a growing commitment from government and other institutions to address the rise in NCDs. In Ghana, funding and research efforts to address the NCD challenge have significantly increased. Between 2019 and 2021, initiatives such as the Measurement Evaluation, Accountability, and Leadership Support (MEALS) for NCD prevention and the Africa Food Environment Research Network (FERN) were launched to support the development and implementation of policies that promote healthy food environments (4).

Currently, Ghana plays a central role in several collaborative projects under the H3Africa Consortium, including the African Wits-INDEPTH partnership for Genomic Studies (AWI-Gen) (7) which aims to understand the interplay between genes, environment, and lifestyle factors in cardiovascular and metabolic health across Africa. Additionally, the Stroke Investigative Research Network (SIREN) aims to identify genomic, sociocultural, economic, and behavioral risk factors for stroke and to build effective research teams to reduce the burden of stroke and other NCDs in sub-Saharan Africa (8). Despite these initiatives, NCDs continue to pose a significant health challenge in Ghana.

Previous research demonstrates that behavior change policies, such as altering physical inactivity and poor dietary habits, can reduce the prevalence of NCDs at the individual and population levels (9). However, to effectively design and develop policies for NCD prevention, it is important to have contextual evidence about the adoption and maintenance of healthier lifestyle behaviors in addition to any potential environmental barriers. This evidence is currently lacking not only in Ghana but also across the broader African context. To address this gap, the Centre for Lifestyle Medicine and Behaviour (CLiMB) at Loughborough University, in collaboration with partners from mainly three Ghanaian institutions the University of Ghana, the Kwame Nkrumah University of Science and Technology (KNUST), and University of Health and Allied Sciences (UHAS) convened a workshop in Ghana during May 2022.

CLiMB at Loughborough University is a world-leading research center of excellence with a track record of implementing impactful lifestyle interventions in the UK. The Centre collaborates with UK research institutions, government public health organizations, such as the National Health Services (NHS), and the UK third-sector organizations. Evidence generated from CLiMB research has influenced policies and practices in the UK and internationally in chronic disease prevention and treatment.

The University of Ghana is a premier academic institution, renowned for its comprehensive research and teaching in various disciplines. It collaborates with global universities and organizations, influencing policies and practices both locally and internationally, particularly in areas such as public health and sustainable development. Kwame Nkrumah University of Science and Technology (KNUST) is a leader in engineering, science, and technology education in Ghana. Known for its strong emphasis on innovation and research, KNUST partners with various industrial and academic institutions worldwide, driving advancements in technology and science across Africa. The University of Allied Health Sciences is specialized in health sciences education and research. It focuses on creating solutions to health challenges through practical and impactful research, collaborating with healthcare institutions and policy-makers to improve public health outcomes in Ghana and beyond.

The key question that the workshop sought to address was: What are the most pressing research gaps in promoting a healthy lifestyle (specifically physical activity and healthy eating) to reduce NCDs in Ghana? Our objective was to discuss and identify research priorities for the prevention of NCDs, with an emphasis on lifestyle behavior change (promoting physical activity and healthier eating), and then to use this information to plan future research studies on this topic. Our collaboration is now subsequently known as CLiMB-Ghana.

## What did we do?

A 2-day hybrid workshop was organized in Accra, Ghana on 28 and 29 May 2023 with the aim of discussing potential research areas and identifying necessary lifestyle interventions in the Ghanaian context to address the increasing prevalence of NCDs. The workshop convened a diverse group of multidisciplinary academic researchers, practitioners, and stakeholders from various institutions from Ghana (University of Ghana, KNUST, University for Development Studies (UDS) and UHAS) from the UK (Loughborough University, University of Sheffield, Bournemouth University, Edge Hill University, University of the West England), Kenya (the Kenyan Technical University). Additionally, academic researchers from France, Netherlands, and the USA, whose research focuses on Ghana, and other African countries and who are interested in lifestyle behaviors, also participated as the expectation is that the collaboration could be extended beyond Ghana to other countries in Africa.

The aims of the CLiMB Ghana initiative are outlines in Box 1. In this report, we provide a summary of the research priorities identified during the workshop. A complete list of workshop attendees and their organizations/institutions is available in the acknowledgements.

BOX 1 **Aims of the CLiMB Ghana initiative.**

•Facilitate collaborations between researchers and practitioners across West and East Africa, initially drawing insights from Ghana and Kenya. Collaborators include counterparts in the Global North, including Europe and North America, specializing in lifestyle medicine and behavior.

•Identify and assess innovative health behavior interventions, focusing on physical activity and dietary behaviors, along with associated policies, aimed at preventing and managing NCDs in Ghana.

•Co-create a comprehensive work plan for the collaboration, emphasizing consensus-building and prioritizing interventions to promote physical activity participation and improve healthy dietary behaviors in Africa, using Ghana as a model country.

•Support capacity building for early and mid-career academics in Ghana interested in NCD prevention through collaborative efforts and knowledge exchange visits.

•Develop and submit grant applications for the development of lifestyle interventions specifically tailored to the African context.

## Research prioritization deliberation process

A total of 35 participants whose research activities aligned with the aims and objectives of the planned collaboration attended the 2-day workshop. The attendees included early to mid-career researchers, practitioners, and senior academics. Policymakers and industry partners were also invited, recognizing the complexity of factors contributing to the rise of NCDs in Africa. The workshop offered a valuable opportunity for learning, sharing experiences, networking, and discussing future research ideas. This was facilitated by presentations of ongoing research in Africa and group discussions among attendees.

Below is a summary of the workshop proceedings:

*Day 1: Overview of participating institutions’ research priorities*

Representatives from each participating institution presented their active research, priorities and strategies related to NCDs. This was followed by discussions to identify overlapping research priorities relevant to the national research agendas of Ghana and Kenya, as the example countries for the initial collaboration. Subsequently, attendants engaged in group activities to identify common research themes and potential areas for collaborative research focusing on lifestyle and NCDs. This process resulted in the identification of four research themes: physical activity, weight management and NCDs, dietary practices and the food environment, methodological and data challenges (see Table 1).

**Table 1:** The research priority topics

**Research priorities**	**Topics**
**Physical activity**	Investigate the influence of the built environment, especially from the perspective of the neighborhood, on walkability, and engage policymakers to incorporate pedestrian walkways into urban planning to support physical activity.
	Assess the current levels of physical activity among various population segments in Ghana.
	Investigate the factors influencing physical activity behaviors and patterns (including cultural and social barriers) and identify strategies for promoting active lifestyles in Ghana.
	Develop evidence-based strategies and recommendations to promote and maintain physically active lifestyles across communities in Ghana, but with a view to adapt for implementation in other African countries
**Weight management and NCDs**	Research using regular screening data for weight management in healthcare facilities.
	Utilise surveillance data from hospital records to monitor weight trends.
	Develop tailored weight loss interventions to suit specific contexts.
**Dietary practices and food environment**	Investigate carbohydrate and energy consumption patterns and employ rigorous measurement methods for carbohydrate intake, to inform dietary interventions for Type 2 diabetes.
	Explore the influence of seasonality on dietary habits and physical activity behaviors.
**Research strategic priorities**	Implement longitudinal and cohort studies to investigate lifestyle behaviors over time.
	Conduct realist reviews to gain insight into the effectiveness of interventions, identifying factors contributing to success or failure and extracting lessons applicable to the local context.
	Utilise available secondary data sources to investigate the burden and epidemiology of non-communicable diseases (NCDs) in Ghana

*Day 2: Research capacity and expertise*

On the second day, the primary objective was to gain insights into participants’ ongoing research projects and to assess the research capacities and expertise of all attendees. Evaluating research skills and expertise is essential for monitoring progress, identifying strengths and weaknesses, and devising a research action plan for the team. Participants’ research expertise spans across disciplines such as food science and nutrition, physical activity, social sciences, communication, immunology, biology, public health, and epidemiology, as well as quantitative and qualitative research methodologies.

## Environmental factors contributing to NCDs in Ghana

Notable challenges in Ghana’s NCD landscape, as highlighted by workshop participants, include food market globalization, the impact of food advertising and the obesogenic environment, physical inactivity due to unsafe neighborhoods, and environmental degradation. Ghana, like other developing nations, grapples with the effects of food market globalization. This trend reflects the growing integration of international food markets into the global economy, leading to a rise in the consumption of processed and unhealthy foods, high in salt, sugar, and unhealthy fats (10). These dietary habits are contributing to the prevalence of NCDs in Ghana.

The obesogenic environment characterised by the proliferation of ultra processed high caloric, low-fibre foods, rapid urbanization, and lifestyle changes, exacerbates the burden of NCDs (11). Concerns also arise from unsafe neighborhoods lacking adequate infrastructure for physical activity, such as pavements and walkways, further limiting opportunities for citizens to be physically active in urban areas.

Moreover, environmental degradation, notably water pollution, poses significant health risks and contributes to the rising prevalence of NCDs. Illegal gold mining activities, known as “galamsey,” heavily contaminates Ghana’s water bodies, with approximately 60% polluted with toxic substances such as mercury and cyanide particularly in the southwestern regions (12). While water pollution directly causes infectious diseases, gastrointestinal issues, and parasitic infections, it also has effect on NCDs. In many LMICs, polluted water sources compel individuals to purchase bottled water or opt for cheaper alternative beverages like sugar-sweetened and carbonated drinks. While some imported bottled water brands can be more expensive than certain fizzy drinks, locally produced bottled water is often more affordable. However in contexts where sugar-sweetened and carbonated drinks are cheaper or more readily available than sage drinking water, individual may be more like to consume them, contributing to increased risk of NCDs such as diabetes and obesity. This situation underscores the complex relationship between environmental issues and public health challenges. Addressing these environmental challenges is crucial to mitigating the NCD burden in Ghana and safeguarding public health.

## Challenges with NCD research

Attendees highlighted several research challenges that may impede the proposed collaboration. Each challenge is detailed below:

- *Funding Constraints:* Researchers in Ghana face limited access to research funding within country, hindering primary studies and essential purchases of research supplies and equipment. This funding scarcity inhibits innovative projects and hampers evidence synthesis to address pressing health issues like NCDs. One strategy currently under consideration in Ghana is the creation of a research fund that allocates 1% of the gross domestic product to support research in the prevention of NCDs and related public health policies (13).
- *Data Deficiency:* Ghana, as is the case in many other African countries, lacks comprehensive NCD-related data, from prevalence to treatment outcomes. This dearth of evidence hampers meaningful analysis and actionable insights, hindering the development of effective intervention and policy formulation. With the limited access to screening and routine checks, the health system also misses early warning systems and data on borderline NCD risks.
- *Ethical Approval Bottlenecks:* Complex administrative procedures can sometimes delay the ethical approval process in Ghanaian institutions. This leads to research timeline setbacks, impeding study progress and output achievement.
- *Limited Institutional Research Capacity:* Building and sustaining research capacity within organizations is hindered by insufficient resources for training of staff and early career researchers, mentorship, and knowledge translation opportunities. This shortfall results in short-lived or unsustainable research projects due to staffing and technical support issues.
- *Policy Disconnect:* Despite the growing NCD burden in Ghana, there is a lack of prioritization of research agendas. The health system is overburdened with curative measures with minimal emphasis on preventative work including research. Consequently, the delayed impact of research findings risks being overlooked by policy makers. While some progress has been achieved, such as the Ghana Health Service’s research agenda alignment with priority areas, a cohesive effort from stakeholders is still lacking and therefore complicating the alignment efforts between funders and researchers.

## Consensus building on research priorities

Workshop attendees agreed on key research priorities through discussions to direct future research in lifestyle medicine and behavior change in Ghana (see Table 1). The top four research priorities relating to NCD prevention are detailed in Table 2.

**Table 2:** Top four important research directions.

**Important research directions**
Conduct population-level observational research across Ghana to explore feasible and acceptable changes which individuals can make to their dietary and physical activity behaviors to facilitate a reduction in NCDS. Undertake a Delphi Study among experts aimed at identifying and prioritising a research agenda concerning NCDs, particularly focusing on lifestyle behaviors. This study will engage researchers, policy makers and programme implementers. Conduct a comprehensive mapping study of interventions, policies, and programmes related to diet and physical activity in Ghana, followed by a realist synthesis to determine their effectiveness, mechanisms, and contextual relevance in Ghana and the broader African context. Evaluate the contextual factors influencing participation in physical activity within the regions of Ghana and Kenya.

The overarching question that guided this discussion was: “What are research priorities for lifestyle behavioral change to reduce the burden of NCDs in Ghana?”

## Addressing research challenges in Africa

Recommendations that emerged from the workshop for addressing the various NCD research challenges included:

- Encourage enhanced collaboration among stakeholders, underpinned by a stakeholder mapping exercise to identify key partners and foster synergy.
- Advocate for government involvement in shaping the research agenda concerning NCDs in Ghana, ensuring alignment with national health priorities.
- Promote interdisciplinary research on NCDs to harness diverse expertise and perspectives, that enrich the depth and breadth of insights generated.
- Re-evaluate the health research agenda in Ghana, particularly examining the impact of macro-level factors such as policies and large-scale food marketing strategies on dietary practices.
- Explore mechanisms for integrating local knowledge into policy formulation and implementation concerning lifestyle interventions, including dietary modifications and physical activity, to ensure cultural relevance and effectiveness.

### Need for a comprehensive approach to NCD research

Workshop attendees commended Ghana’s proactive efforts in NCD-related research and policy. However, they noted that these initiatives often operate independently among various stakeholders, leading to oversight of critical issues and hindering alignment between funders and researchers. The workshop highlighted the need for a holistic approach to research priorities and policies to address the NCD burden in Ghana and beyond. Attendees emphasized the importance of a comprehensive strategy that encompasses research, intervention, and policies to fully understand the complexity of NCDs, create effective interventions, address health inequities, guide policy and practice, and enhance collaborative efforts to improve population health outcomes.

### Adopting a multisectoral and multidisciplinary research approach

The workshop highlighted the need for improved collaboration among stakeholders in Ghana to effectively address NCDs. Despite various organizations working toward this goal, there is still limited active engagement with government ministries, additionally, researchers are often involved in health policy development at later stages, limiting the integration of research findings into policy and practice. To tackle these issues, we propose that future NCD research agenda should be inclusive of the various stakeholders in Ghana.

## Lessons and limitations from the hybrid workshop approach

The hybrid format enabled a mix of local and international perspectives, offering valuable insights from participants with different expertise. This facilitated exchange of best practices and lessons learned from different contexts. By offering both in-person and virtual participation, the workshop was accessible to a broader range of attendees, including those who could not travel. This flexibility facilitated a more inclusive exchange of ideas.

Despite efforts to create a seamless hybrid environment, technical issues (e.g., connectivity problems, audio-visual difficulties) occasionally disrupted the flow of discussions. These challenges were more pronounced for remote participants, who faced delays or difficulties in contributing to real-time discussions, limiting their ability to engage fully.

To enhance future hybrid workshops, it is important to invest in reliable technology and dedicated support to avoid disruptions and to actively facilitate engagement from both in person and remote participants. Furthermore, structured opportunities for interaction, such as having a designated facilitator responsible for engaging online participants can help ensure effective engagement.

## Conclusion

This paper provides an overview identified research priorities, discussions and recommendations from a 2-day hybrid workshop focusing on lifestyle medicine and behavior change research to address NCDs in Ghana. The outcomes of this consultative meeting meaningfully contribute to national efforts to reduce NCD prevalence. It advocates for a multidisciplinary research approach, recognizing the complexity of factors contributing to NCDs, and emphasizes the importance of robust collaboration among stakeholders and active engagement with the public to ensure the relevance of research initiatives. These efforts aim to drive innovative interventions and policies for NCD mitigation and prevention, ultimately promoting the health and well-being of the Ghanaian population, with lessons for other Africans countries.

## Data Availability

The original contributions presented in the study are included in the article/supplementary material, further inquiries can be directed to the corresponding author.

## CLiMB Ghana workshop participants

Humphrey Garti, Victor Mogre, University for Development Studies; Daniel Boateng, Arti Singh, Kwame Nkrumah University for Science and Technology; Reginald Mensah, Richmond Aryeetey, Amos Laar, School of Public Health, University of Ghana; Joy Akameze, Faith Agbozo, Phyllis Addo, University of Health and Allied Sciences; Godfred Egbi, Gloria Folson, Irene Owusu Donkor, Benjamin Abuaku, Dorothea Obiri, Sodey Akoto, Noguchi Memorial Institute for Medical Research; Vida Ayeh, Ama De-Graft Aikins, University College London, Sandra Kushitor, Ensign Global College; Emmanuel Assasie, Vida Nyawornota, Physical Education and Sports studies, University of Ghana; Maame Yaa B Adjei, Justina Serwaah Owusu, Nutrition & Food Science, University of Ghana; Adwoa Pinamang Boateng Desu, World Child Cancer; Labram Musah, Ghana NCD Alliance; Sanda Umar Ismail, University of West England; Paula Griffiths, Emily Rousham, Ines Varela-Silva, Eef Hogervorst, Clare Madigan, Amanda Daley, Hibbah Osei-Kwasi, Loughborough University; Mary Nicolaou, Amsterdam UMC; Sophia Amenyah, Bournemouth university; Andrew Levy, Edge Hill University; Michelle Holdsworth, UMR Moisa; Robert Akparibo, Sheffield University; Brenda Abu, Rochester Institute of Technology.
